# Evaluation of visible diffuse reflectance spectroscopy in liver tissue: validation of tissue saturations using extracorporeal circulation

**DOI:** 10.1117/1.JBO.26.5.055002

**Published:** 2021-05-21

**Authors:** Stylianos Voulgarelis, Faraneh Fathi, Astrid G. Stucke, Kevin D. Daley, Joohyun Kim, Michael A. Zimmerman, Johnny C. Hong, Nicholas Starkey, Kenneth P. Allen, Bing Yu

**Affiliations:** a Medical College of Wisconsin, Children’s Wisconsin, Department of Anesthesiology, Milwaukee, Wisconsin, United States; b Marquette University and Medical College of Wisconsin, Department of Biomedical Engineering, Milwaukee, Wisconsin, United States; c Herma Heart Institute, Children’s Wisconsin, Department of Perfusion, Milwaukee, Wisconsin, United States; d Medical College of Wisconsin, Children’s Hospital of Wisconsin, Department of Surgery, Division of Transplant Surgery, Milwaukee, Wisconsin, United States; e Biomedical Resource Center, Medical College of Wisconsin, Department of Immunology Microbiology, Milwaukee, Wisconsin, United States

**Keywords:** diffuse reflectance spectroscopy, visible-light spectroscopy, liver transplant, tissue saturation

## Abstract

**Significance:** Real-time information about oxygen delivery to the hepatic graft is important to direct care and diagnose vascular compromise in the immediate post-transplant period.

**Aim:** The current study was designed to determine the utility of visible diffuse reflectance spectroscopy (vis-DRS) for measuring liver tissue saturation *in vivo*.

**Approach:** A custom-built vis-DRS probe was calibrated using phantoms with hemoglobin (Hb) and polystyrene microspheres. *Ex vivo* (extracorporeal circulation) and *in vivo* protocols were used in a swine model (n=15) with validation via blood gas analysis.

**Results:** *In vivo* absorption and scattering measured by vis-DRS with and without biliverdin correction correlated closely between analyses. Lin’s concordance correlation coefficients are 0.991 for $\mu_{a}$ and 0.959 for ${\mu_{s}}^{\prime }$. Hb measured by blood test and vis-DRS with ($R^{2}=0.81$) and without ($R^{2}=0.85$) biliverdin correction were compared. Vis-DRS data obtained from the *ex vivo* protocol plotted against the ${\mathrm{PO}}_{2}$ derived from blood gas analysis showed a good fit for a Hill coefficient of 1.67 and $P_{50}=34\;\mathrm{mmHg}$ ($R^{2}=0.81$). A conversion formula was developed to account for the systematic deviation, which resulted in a goodness-of-fit $R^{2}=0.76$ with the expected oxygen dissociation curve.

**Conclusions:** We show that vis-DRS allows for real-time measurement of liver tissue saturation, an indicator for liver perfusion and oxygen delivery.

## 1 Introduction

Non-invasive methods to determine the adequacy of organ perfusion through measurement of tissue oxygen saturation levels are of great clinical interest. Direct measurement of organ saturation allows conclusions regarding the oxygen delivery to and extraction from the tissue. The oxygen delivery depends on the vascular patency, cardiac output, and organ perfusion pressure. Currently, the most widely used method in clinical practice for organ perfusion monitoring is near-infrared spectroscopy (NIRS).^1^^,^^2^ The NIRS tissue penetration depth depends highly on the tissue optical properties and is approximately 1/3 to 1/2 of the source–detector separation distance, i.e., maximally 2 to 3 cm for commercially available devices.^3^^–^^6^ Transcutaneous and direct application of NIRS on the liver have been studied and exhibited wide variability at baseline values and unreliable ability to identify changes in oxygen delivery.^7^

Visible diffuse reflectance spectroscopy (vis-DRS) uses the scattering and absorption of multiple wavelengths in the visible range to determine the optical properties of the underlying tissue, with hemoglobin (Hb) constituting the most important absorber. Vis-DRS has been extensively studied for cancer detection,^5^^–^^20^ monitoring of tumor response to therapy, tumor margin assessment,^18^^–^^27^ and monitoring of tissue damage after thermo-ablation.^28^^,^^29^ Only a few studies so far have used the scattering and absorption properties to determine the ratio of oxygenated to de-oxygenated Hb and thus oxygen saturation in the tissue in acute and chronic mesenteric ischemia,^30^^,^^31^ and in cardiac ^32^ and skeletal muscle tissue,^31^ i.e., in lightly absorptive tissue. The current study investigated whether vis-DRS was suited to determine tissue saturation in highly absorptive liver tissue *in vivo*. In particular, the study was designed to determine the validity of the derived saturation values, the reproducibility of the values with repeated measurements, the effect of the high concentration of biliverdin in the liver on the absorption and scattering, and the ability to determine changes in saturation with changes in oxygen delivery in a “clinical” scenario. We hypothesized that vis-DRS can reliably measure the Hb concentration and the liver tissue oxygenation when applied on the surface of the liver.

## 2 Materials and Methods

Presented is a combination of *in vitro*, *ex vivo* (perfused with cardiopulmonary bypass), and *in vivo* pilot studies that evaluated the ability and validity of vis-DRS to interrogate liver oxygen saturation in a swine model.

### 2.1 Vis-DRS Instrument

**Fig. 1:**
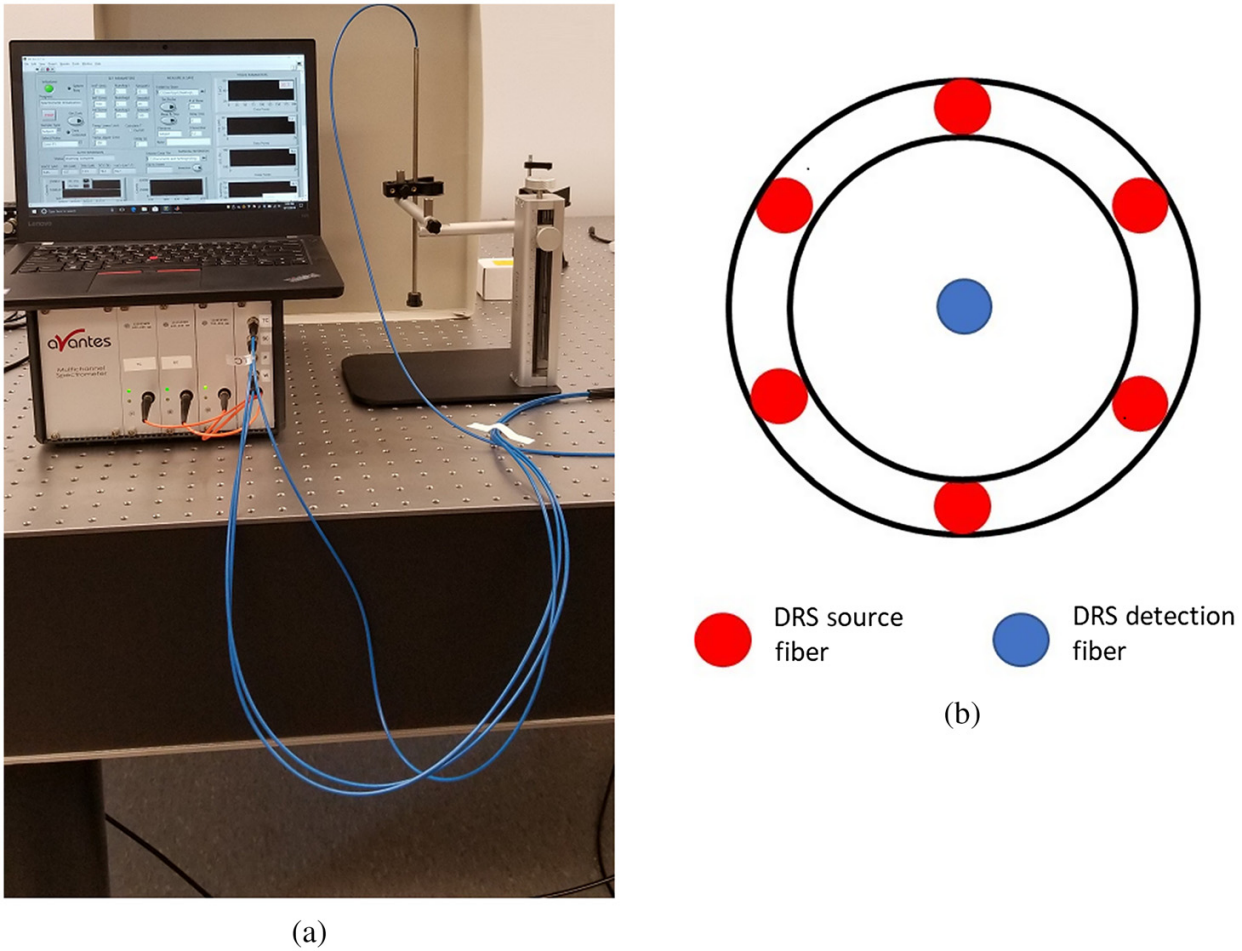
(a) Photograph of the visible diffuse reflectance spectroscopy (vis-DRS) instrument and self-calibration fiber optic surface probe used for the study and (b) diagram of the fiber layout at the distal end of the probe. The center-to-center distance between the illumination ring and detection fiber is 1.2 mm.

The setup, as shown in Fig. 1(a), consists of a self-calibrating fiber optic surface probe and a portable vis-DRS system equipped with a laptop computer for data acquisition.^33^ Our spectroscopy system includes a white-light emitting diode (LED) as the light source and two visible USB-spectrometers (Avantes BV, The Netherland) for spectrum detection. The fiber optic probe is composed of nine 200/220-μm multimode optical fibers, with six for DRS illumination, the seventh one for DRS detection, and the remaining two for self-calibration illumination and detection, respectively. Seven illumination fibers are the highest number that can be included inside a standard 3-mm fiber optic jacket. At the distal end, the six DRS illumination fibers are arranged as a ring around the DRS detection fiber [Fig. 1(b)]. The source-detection separation (SDS), which is the center-to-center distance between the DRS illumination ring and the detection fiber, is 1.2 mm. The SDS is a tradeoff between a large penetration depth and good signal-to-noise ratio in liver tissue. The self-calibration and detections fibers are terminated inside the rigid part of the probe using a diffusely reflective coating material (not shown in Fig. 1).^33^ All illumination fibers are connected to the white LED, and the detection fibers are connected to the two spectrometers, respectively. The real-time diffuse reflectance and self-calibration spectra (430 to 630 nm) are collected by a custom LabVIEW program. For calibration, the tissue spectrum is divided by the self-calibration spectrum collected concurrently to account for real-time instrument drifts and fiber bending loss.^34^ A 3D-printed dark stopper is added to the distal end of the probe to maintain a stable tissue contact while blocking background noise from the room light.

### 2.2 Phantom Validation

Tissue mimicking liquid phantoms were used to evaluate the performance of the instrument in measuring optical properties of highly absorptive liver tissue. The phantoms were created using human Hb powder (H0267, Sigma-Aldrich Co. LLC) as the absorber and 1-μm polystyrene microspheres (07310-15, Polysciences Inc) as the scatterer. The phantoms were obtained through 16 successive titrations of the Hb from 46.36 to 112.01  μM and fixing the number of scatterers. The absorption coefficients ($\mu_{a}$) of the phantoms were independently determined by measuring the $\mu_{a}$ of the Hb stock solution using a spectrophotometer (Lambda35, PerkinElmer) and the Beer–Lambert law. The reduced scattering coefficients (${\mu_{s}}^{\prime }$) were calculated using Mie theory with known particle refractive index, size, and density. Diffuse reflectance and self-calibration spectra in the wavelength range of 435 to 630 nm for each of the 16 phantoms were collected using the fiberoptic probe and spectrometer. Self-calibration was performed by dividing the phantom spectrum by the calibration spectrum (point-by-point). A Monte Carlo inverse model was used to extract the phantom $\mu_{a}$ and ${\mu_{s}}^{\prime }$ and Hb concentration from the calibrated phantom spectrum, following the procedures described by Yu et al.^34^ Finally, the percent errors, which are the difference between the extracted and expected mean values in $\mu_{a}$ and ${\mu_{s}}^{\prime }$, were computed.

### 2.3 Basic Surgical Preparation

The use of animals in this study was approved by the Institutional Animal Care and Use Committees (IACUC) at the Medical College of Wisconsin (MCW) and Zablocki Veterans Affairs Medical Center. The animal use programs at both institutions are fully accredited by the Association for Assessment and Accreditation of Laboratory Animal Care (AAALAC) International. Experiments were carried out on adult (42 to 52 kg) female pigs. The animals fasted for 12 h and were premedicated/sedated with an intramuscular (IM) injection of Tiletamine 3 to 5  mg/kg, Zolazepam 3 to 5  mg/kg, and xylazine 2.2  mg/kg. When the animal was sedated enough, it was taken into the intubation suite. Atropine IM (50  mcg/kg) was given, and an auricular intravenous (IV) catheter was placed prior to intubation. Ocular reflexes were tested for depth of anesthesia, and additional propofol (1 to 2  mg/kg) was administered to achieve appropriate intubating conditions. A single dose of enrofloxacin (5  mg/kg) IM was administered as antibiotic prophylaxis because of the long, invasive surgical preparation. The animal was transferred intubated and ventilated under continuous pulse oximetry to the surgical suite, where they were continuously ventilated using an anesthesia machine (Ohmeda CD, GE, Datex Ohmeda, Madison, Wisconsin). General anesthesia was maintained with 1.5% to 3% isoflurane. Inspiratory oxygen fraction, expiratory carbon dioxide concentration, and expiratory isoflurane concentration were continuously displayed with an infrared analyzer (POET II, Criticare Systems, Waukesha, Wisconsin). An esophageal probe was placed for non-invasive cardiac output (CO) measurements in the initial experiments, where CO values values showed good correlation with blood pressure and pulse pressure amplitude (ECOM, ECOM Medical Inc, San Juan Capistrano, California). The animal was placed in the supine position. The right groin was infiltrated with 1% lidocaine, and femoral venous and arterial catheters were placed through a cut down technique for monitoring, drug administration, and blood sampling. The midline of the abdomen was infiltrated with 1% lidocaine with extension to the subcostal areas bilateral and the sternum before incision. Throughout the experiments, care was taken to increase anesthetic depth for any signs of light anesthesia, e.g., an increase in blood pressure or heart rate. The animal was maintained at 38.5±0.5°C with a warming blanket.

#### 2.3.1 Hemodilution without vascular compromise (*in vivo* hemodilution)

The portal vein or a hepatic vein (depending on the protocol) was identified, and a 22G angiocath was placed with an extension tubing to facilitate blood sampling. Supported by a holder, the vis-DRS probe rested against the liver surface, which allowed free motion during the respiratory cycle without changing the pressure against the tissue. Aliquots of blood were gradually removed through the venous line and stored in a citrated bag, while an isotonic crystalloid solution was used to resuscitate the animal and maintain hemodynamic steady state. Total Hb levels and saturations (${\mathrm{SO}}_{2}=\mathrm{oxy}\text{-}\mathrm{Hb}/\text{total}$ Hb) were obtained with vis-DRS at each Hb concentration and at fractions of inspired oxygen (${\mathrm{FiO}}_{2}$) of 0.21, 0.3, and 1. Blood samples were obtained for analysis during each vis-DRS measurement. All data were recorded using a digital acquisition system and stored on a computerized chart recorder (Powerlab/16SP; ADInstruments, Castle Hill, Australia; LabVIEW, NI, Texas; and MATLAB, The Mathworks, Inc., Natick, Massachusetts).

#### 2.3.2 Perfusion of liver with cardiopulmonary bypass (*ex vivo*)

For this protocol, an auricular arterial line was used for blood pressure monitoring instead of femoral arterial cannulation. The abdominal aorta and the bifurcation into the iliac arteries were identified. 400  Units/kg heparin was given IV for full systemic heparinization. The right iliac artery was cannulated with a 22 to 24F cannula, and the blood was drained to a cardiopulmonary bypass circuit and to an autotransfusion bag. The animal subsequently was euthanized through exsanguination followed by additional IV potassium chloride administration if needed. Euthanasia was confirmed by the absence of pulsatility and end tidal ${\mathrm{CO}}_{2}$. The auricular arterial line was the preferred method of monitoring since the iliac arteries were ligated and used for the cannulation.

After euthanasia, the aortic cannula was flushed with 2 l of Ringer’s lactate solution (LR). The incision was extended to a sternotomy to facilitate the procurement. The portal vein was dissected, cannulated with an 18F cannula, and flushed with 2 l of LR. The hepatic artery was dissected down to the celiac artery and was taken *en bloc* with a part of the abdominal aorta, which was used for cannulation. All collaterals to the stomach and duodenum were carefully ligated. A team from the transplant service including at least one attending surgeon with experience in animal research in the swine model assisted in the dissection, harvest, and cannulation of the liver.

Then the liver was placed in a perfusion container on a rack and perfused through the bypass machine. The cardiopulmonary bypass machine consists of a heart-lung machine (LivaNova Sorin S3, London), oxygenator (LivaNova d101, London), heat exchanger, blood reservoir, and cannulae to produce the flow and pressure through centrifugal force. The hepatic artery and portal vein were perfused through the bypass machine, and the blood was drained passively through the hepatic veins into the perfusion container and then into the bypass reservoir. Blood was at room temperature. The perfusion pressure was monitored through the side port of the hepatic arterial cannula and titrated to a mean of 50 to 55 mmHg. The flow was measured in the initial experiments by placing a vascular probe directly on the hepatic artery (Transonic flow-probe, precision S-series 6 mm, Ithaca, New York). Flow through the hepatic artery and portal vein was adjusted to 1 to 1.2  l/min/kg liver tissue. At this flow, the drop in oxygen partial pressure between pre-hepatic and hepatic venous blood was <2  mmHg as confirmed by blood gas analysis, and consequently, hepatic venous samples were used to determine tissue oxygen saturation. A gallbladder drain was placed and maintained via gravity to decompress the biliary tree and avoid stasis of the highly absorptive bile. An additional 100  Units/kg heparin (of the initial animal weight) was given into the circuit. ${\mathrm{CO}}_{2}$ was added to the oxygenator to maintain pH and ${\mathrm{PCO}}_{2}$ in the blood within the physiologic range (pH 7.2 to 7.4).

Delivered fractional oxygen (${\mathrm{FdO}}_{2}$) was titrated on the cardiopulmonary bypass (CPB) to achieve various Hb saturations. (${\mathrm{FdO}}_{2}$ range of 2% to 100%). Hb was reduced with controlled hemodilution in increments of 3  mg/dl. Vis-DRS was used to determine tissue saturation at each Hb concentration—${\mathrm{FdO}}_{2}$ combination. After achieving steady state, two data sets were obtained, each consisting of 20 measurements. During each measurement, blood samples were taken from the hepatic veins for analysis. Oxygen and ${\mathrm{CO}}_{2}$ partial pressures, pH, glucose, Hb, and electrolytes were determined via blood gas analysis (ABL 800 Flex, Radiometer Copenhagen, Brønshøj, Denmark)

To maintain physiological homeostasis, glucose, potassium, and calcium were closely monitored and supplemented if necessary. All data including hepatic artery perfusion pressure, ${\mathrm{FdO}}_{2}$, ${\mathrm{FdCO}}_{2}$, and temperature were recorded using a digital acquisition system and stored on a computerized chart recorder (Powerlab/16SP; ADInstruments, Castle Hill, Australia).

#### 2.3.3 Protocol of vascular occlusion (*in vivo* vascular occlusion)

After the basic surgical preparation described above (Sec. 2.2.), the stomach was retracted and two stay sutures were placed for better exposure. The biliary duct was dissected, ligated, and divided. A gallbladder drain was placed and kept via gravity. The portal lymph nodes were dissected. The portal vein was identified, and vessel loops were placed around it. The hepatic artery was identified by visualization and palpation and dissected to the celiac trunk. The collateral arteries to the stomach and duodenum were carefully identified and ligated. The portal vein or a hepatic vein (depending on the protocol) was identified and a 22G angiocath was inserted with an extension tubing to facilitate repeated blood sampling. Supported by a holder, the vis-DRS probe rested against the liver surface, which allowed free motion during the respiratory cycle without changing the pressure against the tissue.

A 5-mm vascular occluder was placed around the common hepatic artery. A flow meter was placed on either the common hepatic artery or the left hepatic artery, depending on the anatomy/length of the common hepatic artery. Complete occlusion of the hepatic artery and/or portal vein was obtained using appropriate sizes of bulldog vascular clamps. Heparin 100  Units/Kg IV was administered prior to any vascular occlusion. The administration was repeated every 2 h. Data were obtained before occlusion, immediately after occlusion, 10 min after occlusion and after reperfusion of the vessel, all at ${\mathrm{FiO}}_{2}$ 0.3. All data including blood pressure and airway carbon dioxide concentration were recorded using a digital acquisition system and stored on a computerized chart recorder (Powerlab/16SP; ADInstruments, Castle Hill, Australia; LabVIEW, NI, Texas; and MATLAB, The Mathworks, Inc., Natick, Massachusetts).

### 2.4 Statistical Analysis

The vis-DRS values for tissue scattering and absorption were derived using the Monte Carlo inverse model. A sub-analysis to determine whether the inverse model needed correction for the presence of bile in the liver tissue quantified the extent of agreement between the measurements with and without correction for biliverdin using Lin’s concordance correlation coefficient with standard error adjusted for within-animal replicate measures.^35^^,^^36^ Blood gas values were compared with vis-DRS values calculated with and without correction for biliverdin using the overlapping dependent correlations test of Meng et al.^37^ Analysis showed that correction for biliverdin absorption did not change the values for scattering and absorption in the (physiologic) Hb range of interest, and no correction for biliverdin was used for the other analyses in this study.

Post-hoc data reduction, data plotting and statistical analysis of the pooled data were performed using SigmaPlot 11 (Systat Software, Richmond, California) and R 3.5.0 (R foundation for statistical Computing, Vienna, Austria). Values are expressed as mean±SE or median/ range as appropriate. Statistical significance was assumed for p<0.05. Corrections for multiple comparisons were made within each analysis. The statistical tests for each sub-study are described in the results section.

## 3 Results

A total of 15 animals were used for the various protocols. Whenever possible, animals were used for more than one sub-study.

### 3.1 Phantom Validation

**Fig. 2:**
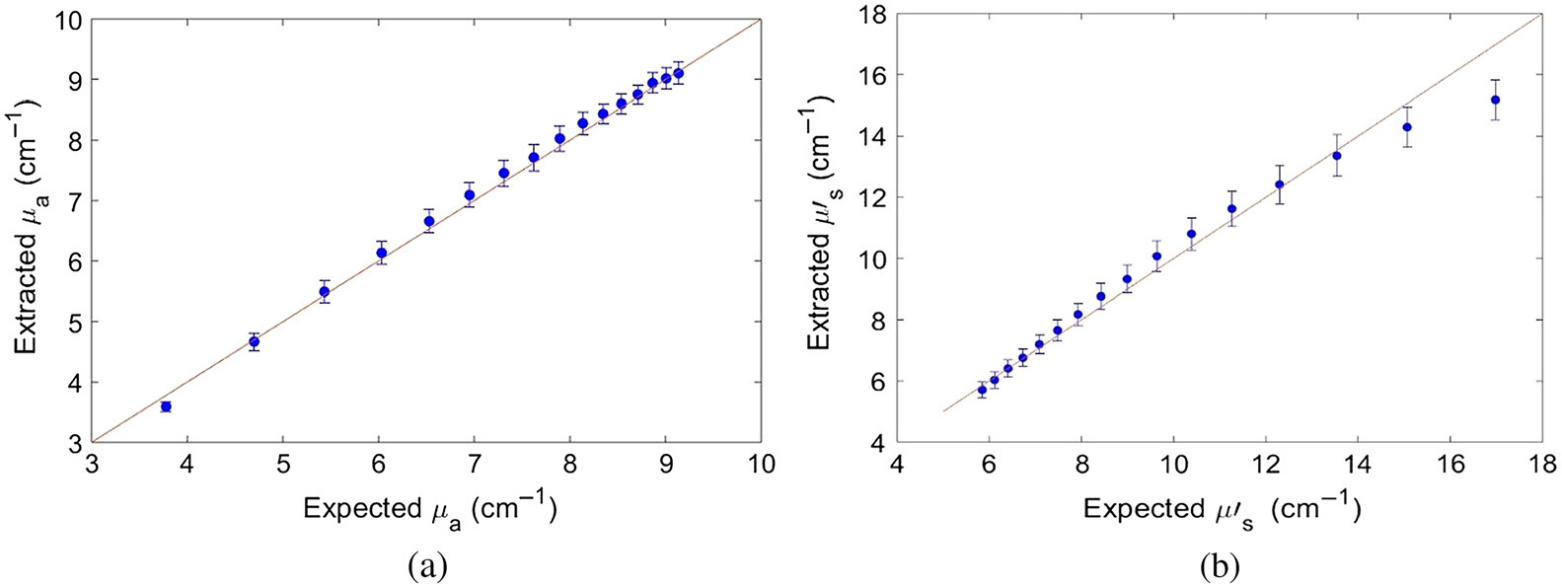
Extracted versus expected phantom mean values for (a) absorption $\mu_{a}$ and (b) scattering ${\mu_{s}}^{\prime }$ from 16 phantom experiments. Mean $\mu_{a}$ phantoms range: 3.87 to $9.35\;{\mathrm{cm}}^{-1}$ with average error of 1.86%. Mean ${\mu_{s}}^{\prime }$ phantoms range: 17.33 to $5.96\;{\mathrm{cm}}^{-1}$ with average error of 4.43%.

The extracted mean $\mu_{a}$ and ${\mu_{s}}^{\prime }$ values of the 16 phantoms are plotted against their expected values in Fig. 2. The extracted mean values and error bars were obtained by averaging over $\mu_{a}$ and ${\mu_{s}}^{\prime }$ values (wavelength averaged) extracted using each of the 16 phantoms as a reference and all phantoms as targets.^34^ The mean $\mu_{a}$ and ${\mu_{s}}^{\prime }$ of the 16 phantoms ranged from 3.87 to $9.35\;{\mathrm{cm}}^{-1}$, and 17.33 to $5.96\;{\mathrm{cm}}^{-1}$, respectively. Average errors of 1.86% and 4.43% were obtained for measuring $\mu_{a}$ and ${\mu_{s}}^{\prime }$, respectively.

### 3.2 Data Variance (*in Vivo* and *ex Vivo*)

Twenty data sets were obtained in three animals using the *in vivo* and *ex vivo* preparation. Each data set consisted of 20 measurements in a 2-min time frame. Data sets were obtained during steady-state conditions and included the whole spectrum of clinically relevant hepatic tissue saturations (29% to 88% for the specific analysis). The coefficient of variation for each data set ranged from 0.7% to 1.6% of the mean value, suggesting that measurement errors were small and independent of the experimental conditions, i.e., Hb concentrations, ${\mathrm{PaO}}_{2}$, hepatic blood flow, or perfusion pressure.

### 3.3 Effects of Correction for the Absorber Biliverdin on Scattering and Absorption

**Fig. 3:**
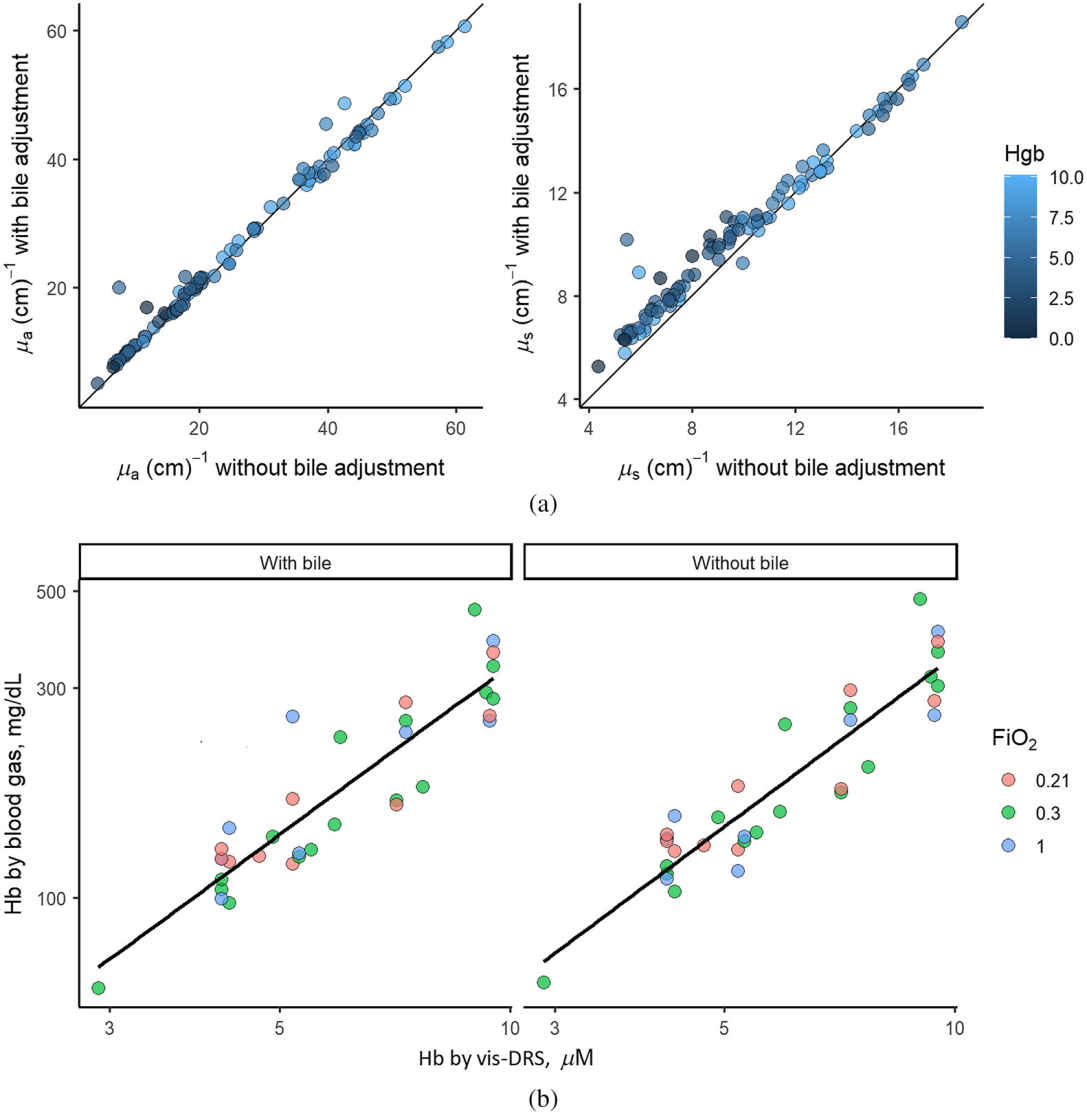
Effects of including biliverdin as an absorption factor in the inverse Monte Carlo model. (a) Values for absorption ($\mu_{a}$, left) and scattering (${\mu_{s}}^{\prime }$, right). Values were obtained from the perfusion of liver with cardiopulmonary bypass (*ex vivo*) protocol. Data points were color-coded for the Hb levels at the time of sampling. Correction for biliverdin resulted in slightly higher ${\mu_{s}}^{\prime }$ values; however, this was not significantly different. Thirty-three data points. (b) Correlation of Hb measured by vis-DRS and blood gas machine (left) with correction for biliverdin and (right) without correction for biliverdin. About 33 data points from three different animals. Data points were color-coded for inspiratory oxygen fraction (${\mathrm{FiO}}_{2}$) at the time of sampling. Data were log-transformed to allow for linear regression analysis. Correction for biliverdin did not significantly affect the Hb values.

Seventy-five data points were obtained from the cardiopulmonary bypass protocol (*ex vivo*) (2.2.2). In general, values for absorption and scattering with and without biliverdin correction correlated closely between analyses. Lin’s concordance correlation coefficients (95% confidence limits) were 0.991 (0.987, 0.994) for absorption and 0.959 (0.943, 0.971) for scattering. The difference between values for absorption and scatter when biliverdin was and was not included as an absorber in the spectral analysis was small at high values and larger at low values (for $\mu_{a}$: $-0.058\pm 0.02\;{\mathrm{cm}}^{-1}$, p=0.009, for ${\mu_{s}}^{\prime }$: $-0.12\pm 0.02\;{\mathrm{cm}}^{-1}$, p=0.0002) [Fig. 3(a)]. For scattering, the difference between values was greater at lower Hb values ($-0.051\pm 0.03\;{\mathrm{cm}}^{-1}$, p=0.047).

### 3.4 Correlation between Blood Gas Analyzer Hemoglobin and vis-DRS Hemoglobin (*in Vivo* Hemodilution)

To evaluate the correlation between the Hb values determined with the vis-DRS method and the values obtained by blood gas analysis, 33 data points were obtained from three animals that underwent the *in vivo* hemodilution protocol to extreme anemia. As shown in Fig. 3(b), there was a close correlation between Hb values measured with the blood gas machine and the values measured with the vis-DRS probe, independent of whether values were corrected for absorption by biliverdin ($R^{2}=0.81$) or not ($R^{2}=0.85$). There was no significant difference between two correlation coefficients (p=0.38), and vis-DRS values with and without correction for biliverdin correlated closely ($R^{2}=0.9$). Due to the excellent correlation between the values for absorption, scattering (3.3.), and Hb (3.4.), we did not include biliverdin as a separate absorber in the Monte Carlo inversion model for the following analyses.

### 3.5 Correlation between Blood Gas Analyzer SO_2_ and vis-DRS SO_2_ (*ex Vivo*)

**Fig. 4:**
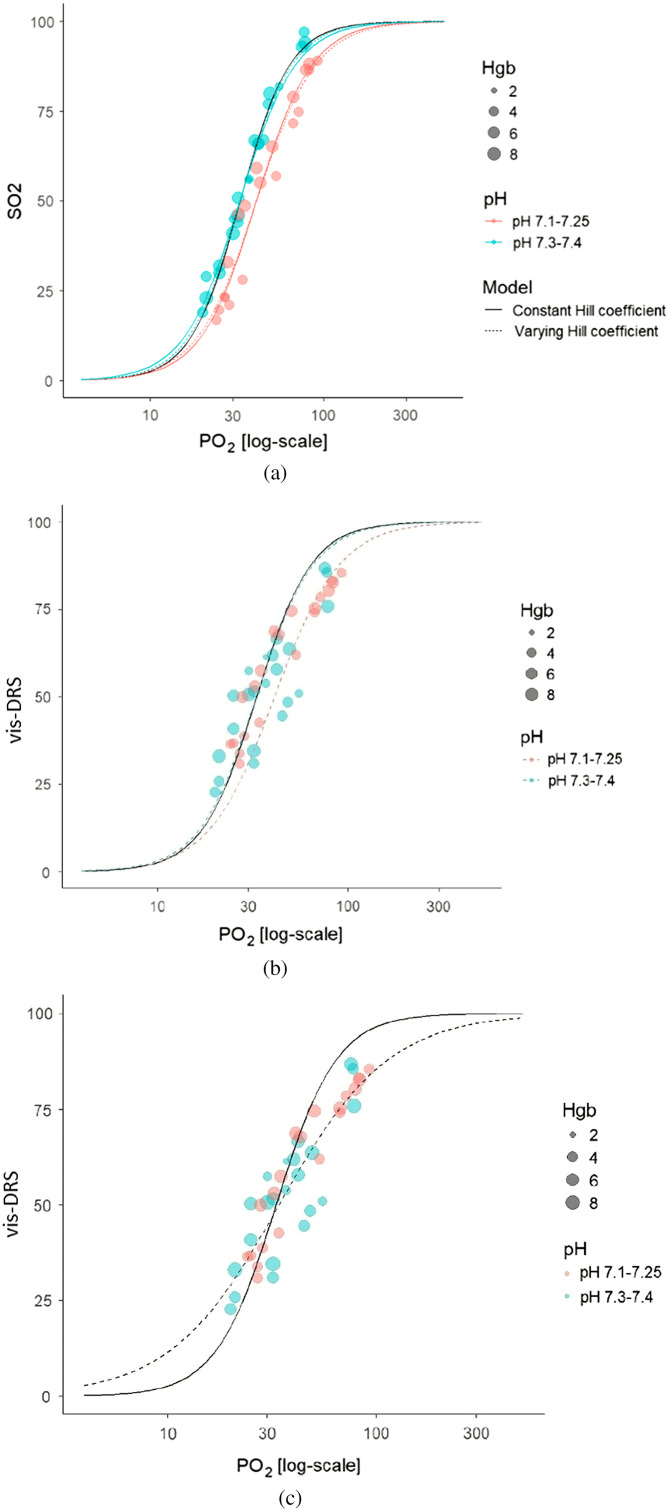
Finding the best oxygen partial pressure (${\mathrm{PO}}_{2}$)—oxygen saturation (${\mathrm{SO}}_{2}$) curve fit for vis-DRS data. (a) Theoretical curve fit: ${\mathrm{SO}}_{2}$ was calculated from the ${\mathrm{PO}}_{2}$ measured with the blood gas machine using the theoretical Hill coefficient of 3.02 and ${\mathrm{PO}}_{2}$ 32.9 mmHg. Goodness-of-fit confirmed excellent fit with the expected Hb-oxygen dissociation curve (black line).^38^ For pooled data: $R^{2}=0.96$ for pH 7.3 to 7.4: $R^{2}=98.1\%$; for pH 7.1 to 7.25: $R^{2}=94.4\%$. (b) Actual curve: vis-DRS values plotted against the same ${\mathrm{PO}}_{2}$ values. The Vis-DRS measurements did not follow the theoretical ${\mathrm{PO}}_{2}/{\mathrm{SO}}_{2}$ curve well but showed a meaningful correlation. (c) Obtaining the best customized curve fit: the vis-DRS data for both pH ranges were pooled. After testing multiple models with varying Hill coefficients and $P_{50}$, we found a good fit with a Hill coefficient of 1.67 and $P_{50}=34\;\mathrm{mmHg}$. The goodness-of-fit was $R^{2}=0.81$. Data points and fitted curves are color-coded for pH; size indicates Hb levels at the time of sampling. Thirty-nine data points.

We used 39 data points from two pig livers perfused via cardio-pulmonary bypass. Values for ${\mathrm{PO}}_{2}$ and calculated ${\mathrm{SO}}_{2}$ obtained from the blood gas samples followed the curve obtained with the Hill equation for pigs with a Hill coefficient of 3.02 and $P_{50}$ of 32.9 mmHg [Fig. 4(a)].^38^

$${\mathrm{SO}}_{2}=\frac{{\left(0.13534\;{\mathrm{PO}}_{2}\right)}^{3.02}}{91.2+{\left(0.13534{\mathrm{PO}}_{2}\right)}^{3.02}}\times 100\%=\frac{{\left({\mathrm{PO}}_{2}/32.929\right)}^{3.02}}{1+{\left({\mathrm{PO}}_{2}/32.929\right)}^{3.02}}\times 100\%.$$ (1)

In Fig. 4(b), we similarly plotted vis-DRS derived values for tissue saturation (within the clinically relevant saturation range of ${\mathrm{SO}}_{2}$ between 20% and 95%) against ${\mathrm{PO}}_{2}$ determined from blood gas analysis. Measurements were performed at two different pH values, adjusted with the addition of ${\mathrm{CO}}_{2}$ (${\mathrm{FdCO}}_{2}$) to the fresh gas in the oxygenator and aimed to reflect pH values typically encountered in human patients (pH 7.15 to 7.4).

The resulting ${\mathrm{PO}}_{2}$-vis-DRS curves did not match the theoretical curve; however, the custom-fitted Hill plot showed a meaningful correlation. Three models were evaluated. Model 1 allowed $P_{50}$ values to vary by pH condition with the Hill coefficient fixed at its value estimated based on the custom fit. This resulted in $P_{50}$ (pH 7.1 to 7.25) = 40.8 mmHg, $P_{50}$ (pH 7.3 to 7.4) = 32.7 mmHg, and Hill coefficient: 2.68. Model 2 allowed both the $P_{50}$ values and the Hill coefficient to vary by pH condition. For pH 7.1 to 7.25, this resulted in $P_{50}=40.9\;\mathrm{mmHg}$ and a Hill coefficient=2.53 and for pH 7.3 to 7.4 in $P_{50}=32.8\;\mathrm{mmHg}$ and a Hill coefficient=2.88. This model had a goodness-of fit of $R^{2}=0.96$ for both datasets. Model 3 used the same $P_{50}$ value and Hill coefficient for both pH conditions, resulting in a $P_{50}=34\pm 1.2\;\mathrm{mmHg}$ and Hill coefficient=1.67±0.15 This model had a goodness-of-fit of $R^{2}=0.81$, which was not significantly different from model 2 (p=0.434) but provided a better fit than the theoretical Hill coefficient (p<0.001). Since in (clinical) practice pH is not continuously measured but generally varies within the range of 7.1 to 7.4, we decided to use the coefficients derived from pooled data for further analysis [Fig. 4(c)].

**Fig. 5:**
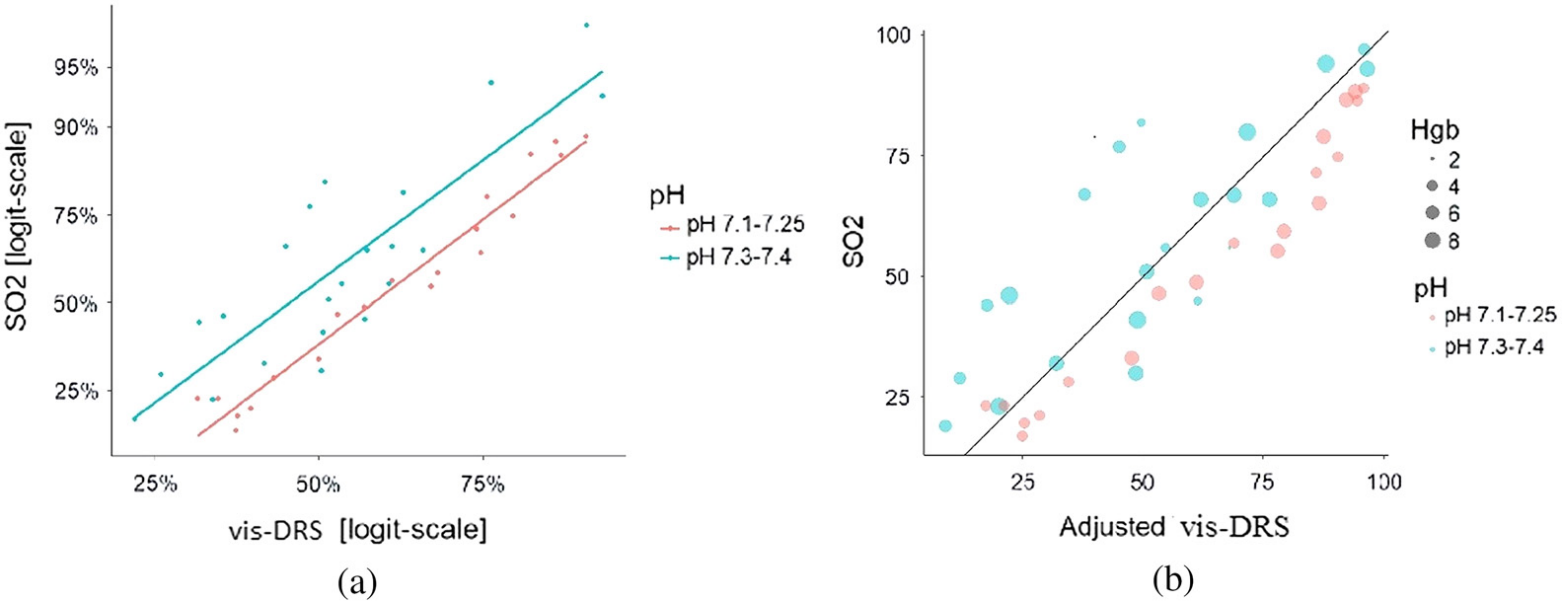
Creating a conversion model for vis-DRS data. (a) ${\mathrm{SO}}_{2}$ and vis-DRS data were logit-transformed to achieve linearity. This allowed the creation of a conversion formula to match the vis-DRS data with the expected ${\mathrm{SO}}_{2}$ values. (see Sec. 3.5). (b) Converted vis-DRS data were plotted against theoretical ${\mathrm{SO}}_{2}$ values. Goodness-of-fit for the pooled data were $R^{2}=0.76$. The data are color-coded for pH; however, the transform was created from the pooled values for both pH levels.

The results suggested that vis-DRS measurements accurately reflected changes in tissue ${\mathrm{PO}}_{2}$ but that a mathematical transform had to be added to the Monte-Carlo inverse model to reflect the true ${\mathrm{SO}}_{2}$ value. Since both ${\mathrm{SO}}_{2}$ and vis-DRS followed the Hill curve, their logit-transformed versions were linearly related [Fig. 5(a)]. This allowed fitting of a conversion model

$${\mathrm{SO}}_{2}=\frac{100}{1+{\left(a\frac{100-\mathrm{Vis}-\mathrm{DRS}}{\mathrm{Vis}-\mathrm{DRS}}\right)}^{r}},$$ (2)

where “r” is the ratio of the respective Hill coefficients of ${\mathrm{SO}}_{2}$ and vis-DRS and α captures the change in $P_{50}$.

We developed an adjustment formula by solving the theoretical Hill equation and the Hill equation fitted to the vis-DRS data or ${\mathrm{pO}}_{2}$. Equating the two equations allowed solving for ${\mathrm{SO}}_{2}$

$${\mathrm{PO}}_{2}/100=32.929\times {\left(\frac{100-{\mathrm{SO}}_{2}}{{\mathrm{SO}}_{2}}\right)}^{1/3.02},$$ (3)

$${\mathrm{PO}}_{2}/100=34.00\times {\left(\frac{100-\mathrm{vis}-\mathrm{DRS}}{\mathrm{vis}-\mathrm{DRS}}\right)}^{1/1.67},$$ (4)

$${\mathrm{SO}}_{2}=\frac{100}{1+1.10{\left(a\frac{100-\mathrm{vis}-\mathrm{DRS}}{\mathrm{vis}-\mathrm{DRS}}\right)}^{1.81}}.$$ (5)

The goodness-of-fit for the correlation between adjusted vis-DRS values and theoretical ${\mathrm{SO}}_{2}$ was $R^{2}=0.76$ [Fig. 5(b)]. We used this conversion to correct all further vis-DRS measurements.

### 3.6 *In Vivo* Vascular Occlusion

**Fig. 6:**
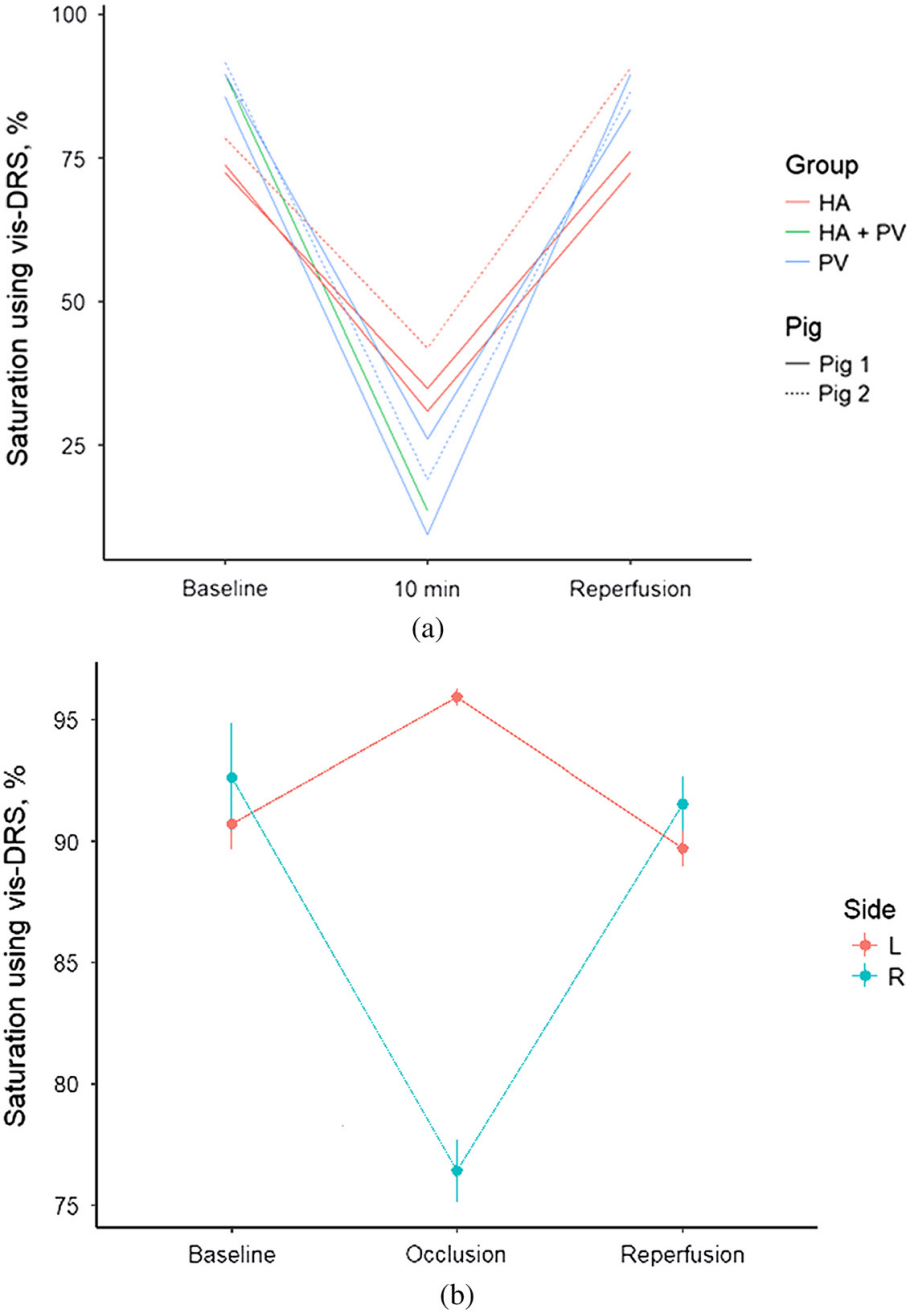
(a) Tissue oxygen saturation as measured by vis-DRS during occlusion of the hepatic artery and/or portal vein. Hepatic artery occlusion: $\Delta_{\mathrm{vis}-\mathrm{DRS}}$: −44.4±6.9%, p<0.001. Portal vein occlusion: $\Delta_{\mathrm{vis}-\mathrm{DRS}}$: −54.3±6.2%, p<0.001. A total of 21 data points were used from two animals. (b) Selective right hepatic artery occlusion shows selective tissue desaturation in the right liver lobe. $\Delta_{\mathrm{vis}-\mathrm{DRS}}-14.7\pm 1.39\%$, p=0.0018. Desaturation promptly recovered when the vessel was reopened. There was no decrease in tissue saturation in the left liver lobe.

We investigated the ability of the vis-DRS method to reliably identify changes in tissue saturation during total occlusion of the hepatic vessels. The analysis included 21 data-points from two animals [Fig. 6(a)].

Vis-DRS was able to identify a decrease in tissue oxygen saturation by 44.4±6.9% from baseline with occlusion of the hepatic artery, which contributes the smaller part of the total oxygen delivery to the liver (p<0.001). Occlusion of the portal vein, which contributes ∼70% of the total oxygen delivery to the liver, reduced vis-DRS by 54.3±6.2% (p<0.001).

To confirm selectivity of this effect to a vascular bed, we selectively occluded the right hepatic artery while the left remained patent. Tissue saturation in the perfusion area of the right hepatic artery (right liver lobe) dropped by 14.7±1.4% (p=0.0018), while saturations slightly increased in the perfused area (left lobe) area ($\Delta_{\mathrm{vis}-\mathrm{DRS}}$: 4.8±1.4%, p=0.04, linear regression model). Saturations returned to baseline after reopening the vessel [Fig. 6(b)].

## 4 Discussion

We used a sequence of phantom, *ex vivo* and *in vivo* experiments to show that vis-DRS is a valid method for determining liver tissue saturation within the physiological range for Hb concentration, oxygen partial pressure, and pH. This study was the first to correlate vis-DRS values with capillary oxygen saturation using extracorporeal circulation to perfuse the liver with blood of known oxygen tension. We observed a systematic deviation of the measured vis-DRS values from the actual tissue oxygen saturation and developed a mathematical conversion formula to correct the values.

### 4.1 Use of Visible Diffuse Reflectance Spectroscopy (vis-DRS) in Low Absorptive Organs *in Vivo*

Vis-DRS reflected changes in myocardial tissue saturation during coronary occlusion in an *in vivo* pig model.^32^ Cardiac tissue saturation was consistently ∼5% higher than coronary venous saturation, which was derived via blood gas sampling. Both values changed similarly with different conditions, suggesting that the tissue saturation measured by the probe correlated with the blood oxygen saturation.^32^ A study using vis-DRS to determine myocardial tissue oxygenation before and after coronary artery bypass surgery in human patients demonstrated wider data variance, which pointed to possible limitations in the interpretation of individual values.^39^ Tissue saturation values could not be validated due to the inability to obtain coronary venous blood samples in human patients. In general, tissue saturations in areas distal to a stenotic coronary artery increased with improved perfusion after bypass surgery. However, baseline tissue saturations proximal to the stenosis were often lower than those in the supposedly ischemic area distal to the stenosis. Vis-DRS values for cardiac tissue in human patients^39^ were substantially lower compared with those measured in healthy pigs.^32^ It is not clear whether this indicated decreased oxygen delivery in hearts with ischemic vascular disease^40^ or differences due to the individual technology.

Vis-DRS has also been used to determine tissue saturation in gastrointestinal mucosa^30^ and muscle in pigs and gastrointestinal mucosa, buccal mucosa, facial skin, and finger muscle in human volunteers and patients.^31^ Muscle tissue saturation in pigs was between 20% and 35% lower than arterial oxygen saturation determined with pulse oximetry. Decreasing ${\mathrm{FiO}}_{2}$ decreased both saturations in a linear fashion and with excellent correlation ($R^{2}=0.98$); however, vis-DRS values decreased more than pulse oximetry. Similarly, vis-DRS values decreased substantially more than pulse oximetry during forefinger ischemia and global ischemia in human subjects. This suggests that pulse oximetry may not be a good method to validate vis-DRS values, in particular, since pulse oximetry overestimates ${\mathrm{SaO}}_{2}$ at values <85% and becomes highly unreliable for ${\mathrm{SaO}}_{2}<66\%$.^40^^,^^41^

### 4.2 Effect of Tissue Homogeneity on vis-DRS Values

Discrepancies between vis-DRS values and tissue saturation values obtained with a second, validated method may also result from the inability to limit the vis-DRS light course to the target tissue. Ubbink et al.^30^ tested the utility of vis-DRS to measure mucosal oxygen saturation of the bowel in pigs *in vivo* by comparing vis-DRS values to oxygen saturation values derived from simultaneously measured microvascular oxygen tension [see Sec. 3.5, Eq. (1)].^38^^,^^41^ While oxygen tension increased with ${\mathrm{FiO}}_{2}$ and decreased promptly after euthanasia, vis-DRS values decreased with increasing ${\mathrm{FiO}}_{2}$ and remained relatively constant after euthanasia, suggesting that the probe measured Hb saturation in submucosal (venous) vessels. In breast tissue, for SDSs spanning 0.23 to 1.10 mm, Bydlon et al.^42^ reported a sensing depth of 0.5 to 2.2 mm. Liver has a much higher Hb content, i.e., absorption is about an order higher than that of breast tissue, but its scatter is about 1/3 to 1/2 of the scattering of breast tissue. For the SDS of 1.2 mm of our custom-made probe, we thus empirically estimated a penetration depth of 0.5 to 1.5 mm below the surface in our model. At this depth, liver tissue is relatively homogenous and without larger blood vessels.^42^ We thus expect that the values obtained with our setup truly reflected the oxygen saturation of the liver tissue.

### 4.3 Effect of Liver Tissue Properties on vis-DRS Values

The absorption and scattering values obtained with vis-DRS in our study [Fig. 3(a)] were quite different from the average values found in the heart muscle, which contains large amounts of myoglobin (average $\mu_{a}∼8\;{\mathrm{cm}}^{-1}$ and ${\mu_{s}}^{\prime }∼12\;{\mathrm{cm}}^{-1}$, Gandjbakhche et al.,^43^ Haggblad et al.^39^) Liver tissue is highly absorptive due to bile that is produced by the organ and that is present intracellularly and in the small bile ducts. We established continuous biliary drainage to avoid biliary stasis and ensure that liver tissue properties remained constant throughout the experiments. Inclusion of biliverdin, the main pigment contained in bile, as an absorber into the Monte Carlo inversions, revealed that inversions that did not include biliverdin slightly underestimated the values for scattering at low levels of scattering and low levels of Hb. This suggests that during extreme hemodilution, i.e., at a low concentration of red blood cells, the tissue properties of the liver including its bile content contributed relatively more to light scattering than at higher levels where scattering was also determined by red blood cells. Gandjbakhche et al.^43^ described for heart muscle that path length and spectral characteristics of the reflected light were greatly dependent on capillary blood content.

In an *in vivo* swine model, bile that was applied to the gut mucosa altered measured vis-DRS values depending on bile composition (Ubbink et al.^30^). We suggest that, in our study, liver tissue properties (very high absorption coefficient but low scattering coefficient compared with other tissues) resulting from bile and high Hb content was responsible for the deviation of the measured vis-DRS values from the standard ${\mathrm{PO}}_{2}\text{-}{\mathrm{SO}}_{2}$ curve for pigs.^38^ This deviation was systematic, and there was a good correlation of vis-DRS values with blood oxygen tension after correcting the vis-DRS values with a conversion formula. Corrected vis-DRS values clearly reflected the acute decrease in tissue oxygen saturation with reduction of blood flow to the liver *in vivo* (Fig. 6). More research is needed to determine whether the conversion formula has to be adapted for specific conditions like fibrosis or cholestasis.

## 5 Conclusion

We have shown that vis-DRS allows for valid, real-time measurement of liver tissue saturation, which is an indicator for liver perfusion and oxygen delivery. Additional mathematical conversion of the original vis-DRS values is required to account for the combined high absorption/low scattering tissue properties of the liver. Vis-DRS is a promising method for promptly diagnosing decreases in liver perfusion, which is an important clinical goal, e.g., to improve graft survival after liver transplantation. *In vivo* studies are required to evaluate the utility of this technique in different clinical scenarios of changing oxygen delivery to the liver.
